# Clinical, Immunological and Inflammatory Characteristics among Mexican Children with Different Subtypes of Juvenile Idiopathic Arthritis: Exploring the Correlation between Anti-Cyclic Citrullinated Peptide (anti-CCP) and Rheumatoid Factor (RF)

**DOI:** 10.3390/pediatric16010014

**Published:** 2024-02-07

**Authors:** Hayde Guadalupe Hernández-Huirache, Dagoberto Armenta-Medina, Edel Rafael Rodea-Montero

**Affiliations:** 1 Department of Pediatric Rheumatology, Hospital Regional de Alta Especialidad del Bajío, León 37544, Mexico; 2 CONAHCyT Consejo Nacional de Humanidades, Ciencias y Tecnologías, Ciudad de México 03940, Mexico; 3 INFOTEC Centro de Investigación e Innovación en Tecnologías de la Información y Comunicación, Aguascalientes 20326, Mexico; 4 Department of Research, Hospital Regional de Alta Especialidad del Bajío, León 37544, Mexico; 5 UPIIG, Instituto Politécnico Nacional, Silao de la Victoria 36275, Mexico

**Keywords:** anti-cyclic citrullinated peptide antibodies, children, juvenile idiopathic arthritis, rheumatoid factor

## Abstract

Introduction: Juvenile idiopathic arthritis (JIA) is the most common chronic rheumatic disease in childhood, affecting one to four of every 1000 children worldwide. It is characterized by joint inflammation lasting more than six weeks in children under 16 years. The aim of this study was to estimate the frequency of JIA subtypes in the Mexican patient population; compare clinical, immunological and inflammation markers by JIA subtype; and examine the correlation between these variables. Methods: We conducted a cross-sectional study of 50 patients with JIA (2–15 years). We estimated the frequency of each JIA subtype, assessed and compared the immunological characteristics (RF, ANA and anti-CCP) by JIA subtype at the time of diagnosis using Kruskal–Wallis or chi-square tests, and calculated Spearman correlation coefficients between the assessments. Results: Our analysis included 50 patients, 29 (58%) girls and 21 (42%) boys, aged at the time of diagnosis 10.56  ±  3.99 years. The frequencies of JIA subtypes were RF-seropositive polyarthritis (34%), RF-seronegative polyarthritis (28%), systemic arthritis (16%), oligoarthritis (14%) and arthritis-related enthesitis (8%). We found a significant association between sex and JIA subtype (*p* = 0.014). There was a significant difference in anti-CCP levels by JIA subtype (*p* < 0.001). We also detected positive correlations between RF and anti-CCP (r = 0.63, *p* < 0.001) and between age and anti-CCP (r = 0.29, *p* = 0.041). Conclusions: Our study suggests that the frequency of the polyarticular subtypes of JIA is higher in Mexican children compared to other populations. Our findings highlight the importance of considering the presence of anti-CCP and RF as important criteria when deciding on treatment for JIA patients as elevated levels of these antibodies may indicate early forms of adult rheumatoid arthritis.

## 1. Introduction

Juvenile idiopathic arthritis (JIA) is the most common chronic rheumatic disease in childhood, but its etiology is unknown. It is characterized by joint inflammation lasting more than six weeks that occurs in children under 16 years and affects one to four of every 1000 children worldwide [1]. There are several classifications of JIA based on its clinical variability, including the following classification groups proposed by the International League Against Rheumatism (ILAR): oligoarthritis; seropositive and seronegative polyarthritis, where seropositive refers to the presence of rheumatoid factor (RF) and seronegative refers to its absence; systemic arthritis; psoriatic arthritis; enthesitis-related arthritis; and other forms of arthritis [2]. To assess and monitor the extent of disease control in JIA, the 27-joint Juvenile Arthritis Disease Activity Score (JADAS-27) was developed and validated [3].

The diagnosis of JIA is made clinically, although there are some biochemical markers that have a certain utility both in the classification of JIA and in its association with extra-articular manifestations. One of these markers is RF, which is found in adult patients with rheumatoid arthritis (RA), but in pediatric patients, its absence does not rule out the disease; therefore, this marker is not useful for diagnosis, although it is useful for the classification of JIA under the ILAR criteria [4,5,6,7]. Another marker is antinuclear antibodies (ANAs), whose presence is associated with the risk of uveitis. ANAs have been observed more frequently in the oligoarticular forms of JIA [8,9]. Patients with JIA developed increased ANA titers and positive RF over the course of the disease [10]. 

An additional marker, anti-cyclic citrullinated peptide antibodies (anti-CCPs), are autoantibodies of great utility in the early diagnosis of RA, with a specificity of up to 98%, and are also useful for predicting erosive forms of the disease in adults [11,12]. Anti-CCPs can be detected in patients with JIA, but their presence is less common than in adult patients with RA [13]. Anti-CCP positivity and bone erosions, the degree of joint damage, and ESR levels are significantly correlated in children with JIA and anti-CCPs could be utilized as a valuable marker in the polyarticular form of JIA [14].

Additionally, compared with non-Hispanic patients, Hispanic patients with JIA have worse scores in terms of disease activity, severity and disability [15]. All of the above make it necessary to study immunological characteristics (RF, ANAs and anti-CCPs) in Mexican pediatric patients with JIA to design treatment programs to avoid the development of erosive complications or deformities.

The objective of this study was to estimate the frequency of JIA subtypes in Mexican patients, compare clinical, immunological and inflammation markers by JIA subtype, and quantify the correlation between variables by sex.

## 2. Patients and Methods

### 2.1. Patients

We conducted a cross-sectional study with a sample of *n* = 50 pediatric patients (29 girls between 2 and 15 years of age and 21 boys between 3 and 15 years of age) who were recruited at the Arthritis Clinic of the Pediatric Rheumatology Department of a Mexican tertiary hospital, Hospital Regional de Alta Especialidad del Bajío (HRAEB), located in the city of León, Guanajuato, Mexico, from February 2009 to December 2019. All participants were Mexican and had a recent diagnosis of JIA classified by subtype according to the ILAR criteria; 2 girls and 6 boys had systemic arthritis, only 4 boys had enthesitis-related arthritis, 4 girls and 3 boys had oligoarthritis, 10 girls and 4 boys had seronegative polyarthritis, and 13 girls and 4 boys had seropositive polyarthritis. No patient presented with the psoriatic JIA subtype.

The exclusion criteria were the simultaneous presentation of JIA and a concomitant disease (heart disease, cancer and/or infection) and having recently undergone surgery, as these clinical conditions can elevate the evaluated acute phase reactants.

### 2.2. Clinical Evaluations

A complete medical history and physical examination were performed by a pediatric rheumatologist at the time of diagnosis, during which age at the time of diagnosis, time from symptom onset to diagnosis, sex, family and disease history, initial symptoms, disease course and progression, and number and characteristics of affected joints and their respective radiological evaluations (joint swelling, osteopenia, joint space narrowing and joint erosions) were collected in order to make diagnoses of JIA and its respective classification by subtype according to the ILAR criteria.

### 2.3. Immunological Evaluations

Blood samples (5–7 mL) were collected at the time of diagnosis by routine venipuncture to perform laboratory tests. These samples were processed in the HRAEB laboratory by personnel without knowledge of the clinical details of the patients. For all assays, the manufacturer’s specifications and recommendations were followed, without modifications.

#### 2.3.1. Rheumatoid Factor (RF)

The presence and concentration of RF in the plasma of patients was measured using a commercial kit (Johnson & Johnson, Raritan, NJ, USA) and processed in a VITROS 5600 integrated system autoanalyzer (Ortho-Clinical Diagnostics, Inc., Johnson & Johnson, Raritan, NJ, USA). Values ≥ 12 IU/mL were considered positive [16].

#### 2.3.2. Antinuclear Antibodies (ANAs)

ANAs were detected using a standard indirect immunofluorescence technique in HEp-2 cells. When ANA titers were ≥1:80 dilution for pediatric samples, the samples were considered positive [17].

#### 2.3.3. Anti-Cyclic Citrullinated Peptide Antibodies (Anti-CCPs)

The presence of anti-CCPs was determined by chemiluminescence using a third-generation commercial anti-CCP test (Abbott, IL, USA) and processed in an Abbott Architect Plus autoanalyzer. When the concentration was ≥20 IU/mL, samples were considered positive [18,19].

#### 2.3.4. Inflammatory Markers: C-Reactive Protein (CRP) and Erythrocyte Sedimentation Rate (ESR)

CRP was determined by immunonephelometry; when the concentration was >0.6 mg/dL, the sample was considered positive [20]. The ESR was determined by the Wintrobe method; when the rate was >10 (mm/h), the result was considered positive [21].

### 2.4. Statistical Analysis

All data were analyzed using the statistical software R [22]. A frequency analysis was performed to determine the frequency of JIA subtypes by sex. Descriptive statistics of the clinical and immunological variables of the study population were calculated. These variables were compared between groups by JIA subtype using the Kruskal–Wallis test or the chi-square test according to the variable type. Lastly, to determine associations, Spearman correlation coefficients were calculated between age and both clinical and immunological variables. The sample size allowed for the detection of differences ≥10% in each of the evaluations (with a type I error of α = 0.05 and a type II error of β = 0.80). For all tests, the level of significance was set to α = 0.05.

## 3. Results

The final analysis included n = 50 patients diagnosed with JIA: 29 (58%) girls and 21 (42%) boys. The mean age at the time of diagnosis ± standard deviation of all patients was 10.56 ± 3.99 years, with a range of 2.13 to 15.10 years. The mean time from symptom onset to diagnosis ± standard deviation of all patients was 2.11 ± 1.81 years. At the time of diagnosis, the average number of active joints was 11.36 ± 6.72, ranging from 1 to 26. Radiological assessments revealed that 74% (37 out of 50) of patients exhibited joint swelling, 70% (35 out of 50) showed osteopenia, 32% (16 out of 50) experienced joint space narrowing and 8% (4 out of 50) displayed joint erosions.

**Figure 1:**
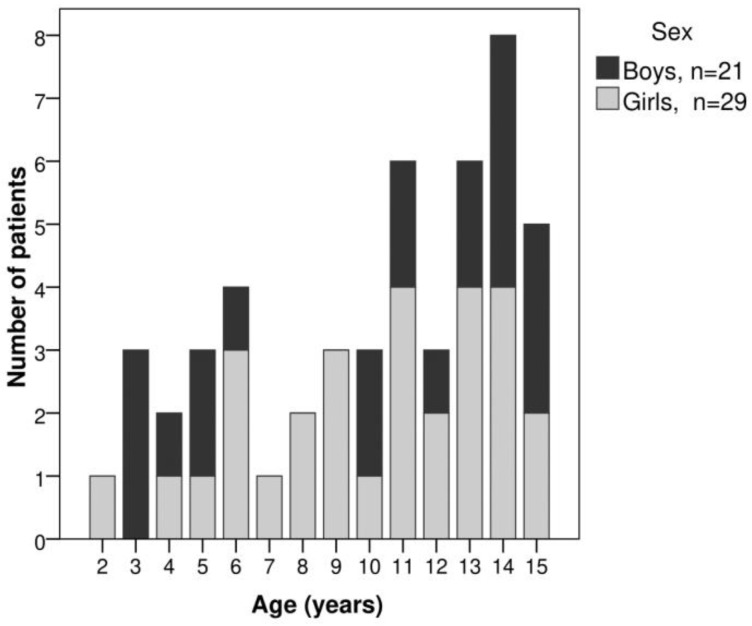
Patients with JIA grouped by age at the time of diagnosis and sex.

**Figure 2:**
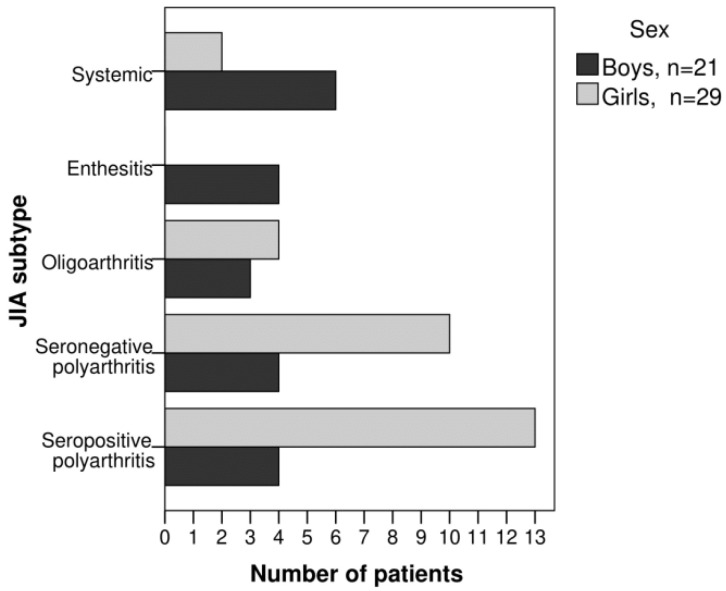
Patients grouped by sex according to JIA subtype.

Figure 1 shows the distribution of patients by age at the time of diagnosis and sex. The patients were divided into five groups based on JIA subtype: 8 (16%) had systemic arthritis, 4 (8%) had enthesitis-related arthritis, 7 (14%) had oligoarthritis, 14 (28%) had seronegative polyarthritis and 17 (34%) had seropositive polyarthritis. No patient presented with the psoriatic JIA subtype. The frequencies of the different JIA subtypes by sex are detailed in Figure 2.

The clinical and immunological characteristics at the time of diagnosis of the study population by JIA subtype are shown in Table 1. Based on intergroup comparisons, a significant association was detected between sex and JIA subtype (*p* = 0.014); systemic arthritis was more frequent in boys (75%), enthesitis-related arthritis occurred only in boys (100%), oligoarthritis was more frequent in girls (57%) and polyarthritis (both seronegative and seropositive) was more frequent in girls (71% and 76%, respectively). The age of patients with enthesitis-related arthritis and seropositive polyarthritis was significantly higher (*p* = 0.007). The number of active joints of patients with polyarthritis (both seronegative and seropositive) was significantly higher (*p* < 0.001). Regarding acute phase reactants, no significant differences were detected in CRP (*p* = 0.348) or ESR (*p* = 0.225) among the different JIA subtypes. 

**Figure 3:**
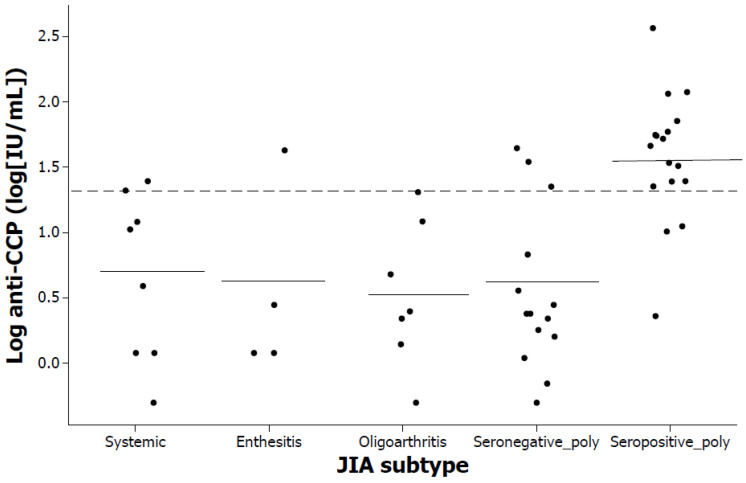
Log anti-CCP values in patients with different JIA subtypes. Solid horizontal lines indicate the means of the data associated with each JIA subtype. The cutoff point is indicated by the dotted line (20 IU/mL).

Tests for RF were negative not only in patients with seronegative polyarthritis and enthesitis-related arthritis but also in all those with the systemic arthritis and oligoarthritis subtypes. A significant difference was detected in anti-CCP levels by JIA subtype (*p* < 0.001); anti-CCP levels were higher in patients with seropositive polyarticular JIA. A significant association was identified between JIA subtype and positive anti-CCP levels (*p* < 0.001); in descending order by JIA subtype, the percentage of patients who had positive values (anti-CCPs ≥ 20 IU/mL) was 82% of patients with seropositive polyarthritis, 25% of patients with systemic arthritis, 25% of patients with enthesitis-related arthritis, 21% of patients with seronegative polyarthritis and 14% of patients with oligoarthritis. The log anti-CCP values in patients by JIA subtype are detailed in Figure 3. Regarding ANAs, only 2% of the sample was positive, that is, only one in 50 patients who presented with seropositive polyarthritis. 

A positive and statistically significant Spearman correlation was observed between anti-CCPs and number of active joints when considering all patients (r = 0.340, *p* = 0.016). Additionally, a positive but not statistically significant Spearman correlation was found between age and number of active joints for all patients (r = 0.243, *p* = 0.089). Furthermore, Table 2 presents the Spearman correlation coefficients (r) calculated for clinical and immunological variables, considering all patients and stratified by sex. CRP was positively and significantly correlated with ESR (r = 0.38, *p* = 0.008) when considering all patients, but, when analyzed by sex, this correlation only remained significant for boys (r = 0.50, *p* = 0.029). Age was moderately and significantly correlated with RF in all patients (r = 0.40, *p* = 0.004), but, when analyzed by sex, this significance was only maintained for girls (r = 0.51, *p* = 0.005). A positive and significant correlation between anti-CCPs and age was also detected when considering all patients (r = 0.29, *p* = 0.041). 

**Figure 4:**
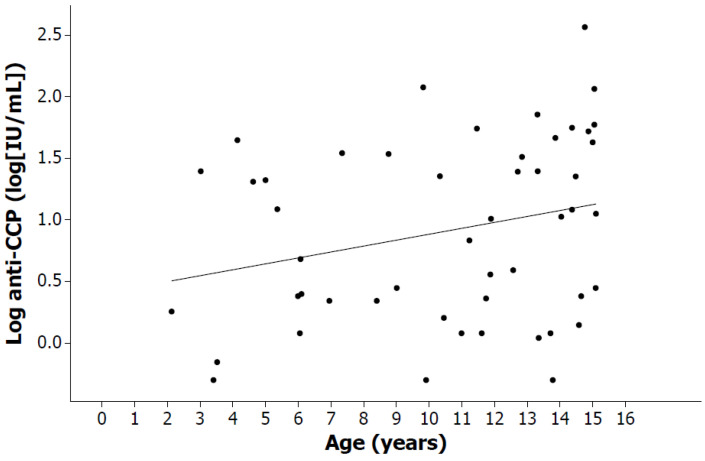
Positive correlation between age and log anti-CCP titers (r = 0.29, *p* < 0.041), considering all patients.

**Figure 5:**
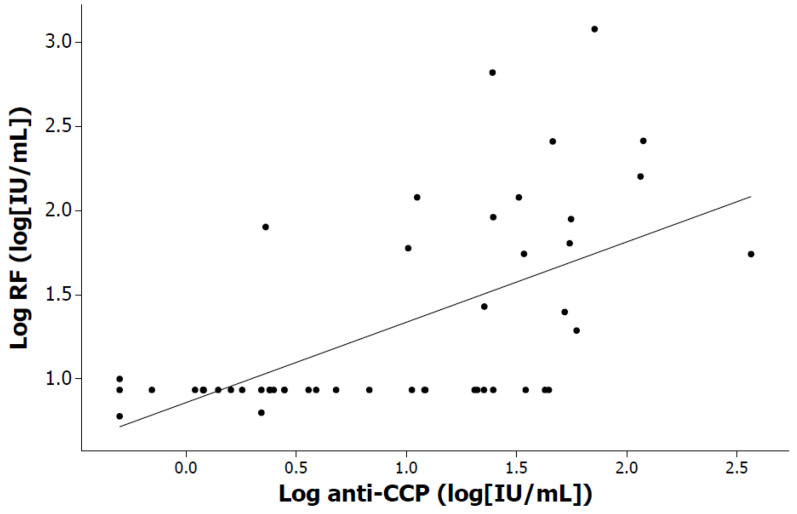
Positive correlation between log anti-CCP titers and log RF (r = 0.63, *p* < 0.001), considering all patients.

Moreover, Figure 4 shows the correlation between age and log anti-CCPs (r = 0.29, *p* = 0.041), considering all patients. In addition, a positive correlation was identified between RF and anti-CCPs when considering all patients (r = 0.63, *p* < 0.001), and this significance was preserved for both girls and boys when analyzed by sex (r = 0.60, *p* < 0.001 and r = 0.58, *p* = 0.006, respectively). Subsequently, Figure 5 shows the correlation between log anti-CCPs and log RF (r = 0.63, *p* < 0.001), considering all patients.

Lastly, patients were categorized into three groups depending on their positivity for RF or anti-CCPs (both negative, only one positive, both positive). The clinical characteristics of the study population at the time of diagnosis are presented in Table 3. Patients’ age and sex distribution were similar between the three groups (*p* = 0.067 and *p* = 0.139, respectively). A statistically significant difference for the variable number of active joints was detected (*p* = 0.007), with higher values observed in patients positive for both RF and anti-CCPs. According to the radiological assessments and intergroup comparison, joint swelling and osteopenia were similar between the three groups (*p* = 0.884 and *p* = 0.118, respectively). However, joint space narrowing was more prevalent in those positive for both RF and anti-CCPs (*p* = 0.007). Although the group that was positive for both RF and anti-CCPs displayed a higher presence of joint erosions, this difference was not statistically significant (*p* = 0.493)

## 4. Discussion

Our findings expand the understanding of JIA in Mexican children, revealing a higher frequency of polyarticular subtypes compared to other populations, with females predominantly affected by these subtypes. In addition, we found that anti-CCPs occur in a greater proportion of patients with RF-positive polyarthritis than in patients with other subtypes of JIA. Furthermore, our analyses demonstrate a positive correlation between age and the presence of RF and anti-CCPs in JIA patients and a positive correlation between RF and anti-CCPs in this cohort.

In our study involving 50 JIA patients, we observed a higher frequency of JIA in girls, similar to that reported by Omar et al. [23] in a multicenter study that evaluated 54 patients with JIA. Regarding age at the time of diagnosis, we identified six patients who were under the age of five years. This finding indicates that it is possible to diagnose JIA at an early age in our population, potentially favoring early treatment and the absence of erosive complications, as suggested by Yasui et al. [24], who reported the case of a very young girl (3 years and 5 months) with seropositive polyarticular JIA and anti-CCP positivity who was successfully treated with tocilizumab. In our sample of the Mexican population, there was a predominance of females with the oligoarticular and polyarticular (both seropositive (RF ≥ 12 IU/mL) and seronegative (RF < 12 IU/mL)) forms of arthritis, with the systemic and enthesitis-related arthritis forms more predominant in males, in agreement with what has been described in the Spanish population [25].

Table 4 details the frequency of JIA subtypes based on the ILAR criteria of diverse studies included for the review of this topic. We observed a notably high frequency of polyarthritis (both seropositive and seronegative) in 62% of the Mexican population, a figure distinct from those reported in other countries. Adib et al. [21] reported a frequency of 18.7% for polyarthritis (both seropositive and seronegative) in the UK population, while Modesto et al. [26] estimated a frequency of 12.4% in the Spanish population. Kunjir et al. [27] found a frequency of 29.0% in the Indian population, Weakley et al. [28], reported 41.0% in the South African population and Tebo et al. [29] estimated a frequency of 31.7% in the American population. Moreover, in studies from the UK, Spain, South Africa and the US, the oligoarticular form was the most common; however, in our study sample, only 14% of patients had this JIA subtype.

The presence of anti-CCPs has been identified in a greater proportion of patients with seropositive polyarthritis than in patients with other subtypes of JIA [30], even at very early ages [24]. In agreement, in our study, positive anti-CCP values (≥20 IU/mL) were found in 82% of patients with seropositive polyarthritis, 25% of patients with systemic arthritis, 25% of patients with enthesitis-related arthritis, 21% of patients with seronegative polyarthritis and 14% of patients with oligoarthritis. We also found that anti-CCPs were detected mainly in patients with seropositive polyarticular JIA whose age was significantly older than that of patients in other groups (*p* = 0.007), confirming what was described by Dewint et al. [31]. This finding is important because it has been found that anti-CCPs are detected more frequently in patients with JIA with severe manifestations, such as the presence of erosions and deformities [32,33], and it has also been identified that anti-CCPs are positively correlated with age [31]. In our sample of Mexican children, we observed that age was positively correlated with RF (r = 0.40, *p* = 0.004) and anti-CCPs (r = 0.29, *p* = 0.041), possibly because of the similarity with adult arthritis. We also identified a positive correlation between RF and anti-CCPs (r = 0.63, *p* < 0.001). Studies in other populations have evaluated the role of anti-CCPs in JIA and have also found anti-CCPs mainly in patients with polyarticular arthritis and RF [13,34].

In addition, despite the absence of JADAS-27, our clinical observations revealed that patients positive for both RF and anti-CCPs exhibited a higher number of active joints (*p* = 0.007). Furthermore, radiological findings indicated a greater prevalence of joint space narrowing in individuals positive for both RF and anti-CPPs (*p* = 0.007). These results align with several studies suggesting that the presence of anti-CCPs in JIA is associated with a worse prognosis compared to forms of JIA where anti-CCPs are absent [32,35]. Anti-CCPs can be useful as a marker of JIA severity, allowing for early therapeutic intervention [23,36], and there is growing evidence that early therapeutic intervention in the course of JIA leads to better disease control and a better prognosis [37]. Therefore, early biological treatment of children with RF and anti-CCPs may be an optimal strategy [24].

This study has some limitations. Firstly, it was a cross-sectional study and, therefore, it is not possible to infer causality. Secondly, the findings are based on a single-center study with a small number (n = 50) of patients with JIA who were not recruited from the general population. They were referred to our center because they were identified as a high-risk population in primary and secondary care centers; therefore, the findings should be interpreted with care. Thirdly, this study was constrained by the lack of a disease activity score, such as JADAS-27, to comprehensively evaluate the disease severity. Lastly, there was no control group, but the objective of this study was to estimate the frequency of JIA subtypes in Mexican patients. The greatest strength of this study is that it allowed for characterizing and comparing immunological variables by JIA subtype to identify and focus on Mexican children who are at a greater risk of complications and thus design effective management and treatment measures.

In Mexico, the overall frequency of JIA and its distribution by subtype remain unknown. Therefore, multicenter studies are required to help accurately estimate the frequency of JIA and its distribution by subtype in the Mexican population. In addition, follow-up studies that include disease activity scores are required that accurately evaluate immunological parameters, radiological changes and the use of biologic agents to prevent erosions and sequelae. These studies can provide new findings regarding the progression of JIA and facilitate the identification of the most effective therapies for these children.

## 5. Conclusions

The results from our study indicate that the frequency of the seropositive and seronegative polyarticular subtypes of JIA in Mexican children is higher than in other populations, with females predominantly affected by these subtypes. There is also a predominance of females affected by the oligoarticular subtype, but, for the systemic and enthesitis-related arthritis subtypes, males are predominantly affected. Additionally, anti-CCPs occur in a greater proportion of patients with seropositive polyarthritis than in patients with other subtypes of JIA.

Our data suggest that age is positively correlated with RF and anti-CCPs in patients with JIA and that there is a positive correlation between RF and anti-CCPs in these patients. Elevated anti-CCPs and RF were associated with a higher number of active joints and a greater prevalence of joint space narrowing and appear to be early forms of adult RA. This finding indicates that the presence of anti-CCPs and RF constitutes an important criterion to consider when deciding on treatment in this population. Nevertheless, it is necessary to further study these patients to predict the course of their disease and design the best treatment scheme that minimizes the sequelae of each JIA subtype. It is necessary to conduct multicenter studies with larger sample sizes that support the findings of the present study and favor the design of treatments in the medium term and long term.

## Figures and Tables

**Table 1:** Clinical and immunological characteristics of the study population at the time of diagnosis by JIA subtype.

Variables	All Patients (n = 50)		Systemic Arthritis (n = 8)	Enthesitis-Related Arthritis (n = 4)	Oligoarthritis (n = 7)	Seronegative Polyarthritis (n = 14)		Seropositive Polyarthritis (n = 17)		Intergroup Comparison
Sex										*p* = 0.014 ^a,c^
girls, n (%)	29 (58%)		2 (25%)	0 (0%)	4 (57%)	10 (71%)		13 (76%)		
boys, n (%)	21 (42%)		6 (75%)	4 (100%)	3 (43%)	4 (29%)		4 (24%)		
age (years)	10.56 (3.99)		8.76 (4.86)	13.70 (1.91)	7.66 (3.49)	9.31 (4.21)		12.9 (1.98)		*p* = 0.007 ^b,c^
active joints	11.36 (6.72)		8.25 (3.62)	6.50 (1.29)	2.43 (0.98)	15.00 (7.64)		14.65 (3.95)		*p* < 0.001 ^b,c^
Radiological assessments										
joint swelling, n (%)	37 (74%)		7 (88%)	4 (100%)	7 (100%)	6 (43%)		13 (76%)		*p* = 0.019 ^a,c^
osteopenia, n (%)	35 (70%)		4 (50%)	1 (25%)	3 (43%)	13 (93%)		14 (82%)		*p* = 0.014 ^a,c^
joint space narrowing, n (%)	16 (32%)		0 (0%)	0 (0%)	0 (0%)	6 (43%)		10 (59%)		*p* = 0.004 ^a,c^
joint erosions, n (%)	4 (8%)		0 (0%)	0 (0%)	0 (0%)	2 (14%)		2 (12%)		*p* = 0.604 ^a^
CRP (mg/dL)	28.6 (43.49)	n = 47	70.28 (76.16)	10.15 (14.31)	11.19 (8.78)	16.56 (16.86)	n = 12	29.04 (39.21)	n = 16	*p* = 0.348 ^b^
ESR (mm/hr)	25.94 (18.64)	n = 47	28.50 (20.00)	8.75 (5.12)	27.71 (13.31)	27.75 (17.43)	n = 12	26.81 (22.19)	n = 16	*p* = 0.225 ^b^
RF (IU/mL)	72.52 (194.00)		8.28 (0.92)	8.60 (0.00)	8.47 (1.09)	8.6 (0.00)		196.82 (300.65)		*p* < 0.001 ^b,c^
<12 (negative), n (%)	33 (66%)		8 (100%)	4 (100%)	7 (100%)	14 (100%)		0 (0%)		*p* < 0.001 ^a,c^
≥12 (positive), n (%)	17 (33%)		0 (0%)	0 (0%)	0 (0%)	0 (0%)		17 (100%)		*p* < 0.001 ^a,c^
log RF, (log [IU/mL])	1.29 (0.58)		0.91 (0.06)	0.93 (0.00)	0.92 (0.06)	0.93 (0.00)		2.01 (0.48)		*p* < 0.001 ^b,c^
anti-CCPs (IU/mL)	27.97 (56.12)		9.41 (9.44)	11.95 (20.45)	6.29 (7.36)	9.11 (14.17)		64.94 (84.49)		*p* < 0.001 ^b,c^
<20 (negative) n (%)	29 (58%)		6 (75%)	3 (75%)	6 (86%)	11 (79%)		3 (18%)		*p* < 0.001 ^a,c^
≥20 (positive) n (%)	21 (42%)		2 (25%)	1 (25%)	1 (14%)	3 (21%)		14 (82%)		*p* < 0.001 ^a,c^
log anti-CCPs, (log [IU/mL])	0.91 (0.73)		0.66 (0.64)	0.56 (0.73)	0.52 (0.55)	0.54 (0.60)		1.58 (0.49)		*p* < 0.001 ^b,c^

Unless otherwise indicated, the values are expressed as the mean (standard deviation). ^a^ Chi-square test with 4 degrees of freedom. ^b^ Kruskal–Wallis test with 4 degrees of freedom. ^c^ Significant *p*-values.

**Table 2:** Spearman correlation coefficients (r) for clinical and immunological variables.

Correlations	CRP		ESR		RF		Anti-CCPs	
	r	significance	r	significance	r	significance	r	significance
Age								
All	0.05	*p* = 0.731	−0.14	*p* = 0.339	**0.40**	*p* = 0.004 ^a^	**0.29**	*p* = 0.041 **^a^**
Girls	−0.10	*p* = 0.612	−0.27	*p* = 0.159	**0.51**	*p* = 0.005 ^a^	0.32	*p* = 0.086
Boys	0.26	*p* = 0.287	−0.08	*p* = 0.745	0.35	*p* = 0.124	0.30	*p* = 0.180
CRP								
All			**0.38**	*p* = 0.008 ^a^	0.05	*p* = 0.734	−0.04	*p* = 0.767
Girls			0.36	*p* = 0.058	0.12	*p* = 0.552	0.04	*p* = 0.832
Boys			**0.50**	*p* = 0.029 ^a^	−0.02	*p* = 0.932	−0.22	*p* = 0.373
ESR								
All					<0.01	*p* = 0.997	0.11	*p* = 0.482
Girls					0.03	*p* = 0.866	0.04	*p* = 0.853
Boys					−0.17	*p* = 0.494	0.13	*p* = 0.603
RF								
All							**0.63**	*p* < 0.001 **^a^**
Girls							**0.60**	*p* < 0.001 ^a^
Boys							**0.58**	*p* = 0.006 ^a^

In all cases, 50 patients (29 girls and 21 boys) were considered, except for the variables CRP and ESR, for which 47 patients were considered (28 girls and 19 boys). ^a^ Significant *p*-values.

**Table 3:** Clinical characteristics at the time of diagnosis in the study population (n = 50), stratified by positivity for RF or anti-CCPs.

Variables	RF and Anti-CCPs Negative n = 26	Either RF or Anti-CCPs Positive n= 10	RF and Anti-CCPs Positive n = 14	Intergroup Comparison
Sex				
girls, n (%)	14 (54%)	4 (40%)	11 (79%)	*p* = 0.139 ^a^
boys, n (%)	12 (46%)	6 (60%)	3 (21%)	*p* = 0.139 ^a^
age (years)	9.82 (4.01)	9.23 (4.90)	12.89 (2.06)	*p* = 0.067 ^b^
active joints	9.69 (7.58)	10.70 (5.70)	14.93 (4.16)	*p* = 0.007 ^b,c^
Radiological assessments				
joint swelling, n (%)	20 (77%)	7 (70%)	10 (71%)	*p* = 0.884 ^a^
osteopenia, n (%)	15 (60%)	9 (90%)	11 (79%)	*p* = 0.118 ^a^
joint space narrowing, n (%)	4 (15%)	3 (30%)	9 (64%)	*p* = 0.007 ^a,c^
joint erosions, n (%)	1 (3.8%)	1 (10%)	2 (14.3%)	*p* = 0.493 ^a^

Unless otherwise indicated, the values are expressed as the mean (standard deviation). ^a^ Chi-square test with 2 degrees of freedom. ^b^ Kruskal–Wallis test with 2 degrees of freedom. ^c^ Significant *p*-values. RF positive: RF ≥ 12 IU/mL, anti-CCP positive: anti-CCP ≥ 20 IU/mL.

**Table 4:** Epidemiological studies examining the frequency of juvenile idiopathic arthritis subtypes based on the International League Against Rheumatism criteria.

				Percentage						
Source	Country	n	Girls/Boys	Systemic Arthritis	Enthesitis-Related Arthritis	Oligoarthritis	Psoriatic	Undifferentiated Arthritis	Seronegative Polyarthritis	Seropositive Polyarthritis
Adib et al. 2008 [21]	UK	427	65%/35%	6.3%	7.5%	54.6%	8.2%	4.7%	15.9%	2.8%
Modesto et al. 2010 [26]	Spain	145	64%/36%	6.9%	12.4%	51.0%	6.2%	11.1%	10.3%	2.1%
Kunjir et al. 2010 [27]	India	235	42%/58%	8.0%	36.0%	21.0%	1.0%	5.0%	17.0%	12.0%
Weakley et al. 2012 [28]	South Africa	78	50%/50%	7.7%	23.0%	27.0%	1.3%	0.0%	26.9%	14.1%
Tebo et al. 2012 [29]	US	334	66%/34%	9.3%	8.1%	43.4%	0.0%	7.5%	22.7%	9.0%
Hernández-Huirache et al., 2024	Mexico	50	58%/42%	16.0%	8.0%	14.0%	0.0%	0.0%	28.0%	34.0%

## Data Availability

All data underlying the findings are available upon request to the corresponding author (edel.rodea@hraeb.gob.mx).
